# Exploration of Flavonoids as Lead Compounds against Ewing Sarcoma through Molecular Docking, Pharmacogenomics Analysis, and Molecular Dynamics Simulations

**DOI:** 10.3390/molecules28010414

**Published:** 2023-01-03

**Authors:** Muhammad Yasir, Jinyoung Park, Eun-Taek Han, Won Sun Park, Jin-Hee Han, Yong-Soo Kwon, Hee-Jae Lee, Mubashir Hassan, Andrzej Kloczkowski, Wanjoo Chun

**Affiliations:** 1 Department of Pharmacology, Kangwon National University School of Medicine, Chuncheon 24341, Republic of Korea; 2 Department of Medical Environmental Biology and Tropical Medicine, Kangwon National University School of Medicine, Chuncheon 24341, Republic of Korea; 3 Department of Physiology, Kangwon National University School of Medicine, Chuncheon 24341, Republic of Korea; 4 College of Pharmacy, Kangwon National University School of Medicine, Chuncheon 24341, Republic of Korea; 5 The Steve and Cindy Rasmussen Institute for Genomic Medicine at Nationwide Children’s Hospital, Columbus, OH 43205, USA

**Keywords:** Ewing sarcoma, flavonoids, molecular docking, molecular dynamics simulations

## Abstract

Ewing sarcoma (ES) is a highly malignant carcinoma prevalent in children and most frequent in the second decade of life. It mostly occurs due to t(11;22) (q24;q12) translocation. This translocation encodes the oncogenic fusion protein EWS/FLI (Friend leukemia integration 1 transcription factor), which acts as an aberrant transcription factor to deregulate target genes essential for cancer. Traditionally, flavonoids from plants have been investigated against viral and cancerous diseases and have shown some promising results to combat these disorders. In the current study, representative flavonoid compounds from various subclasses are selected and used to disrupt the RNA-binding motif of EWS, which is required for EWS/FLI fusion. By blocking the RNA-binding motif of EWS, it might be possible to combat ES. Therefore, molecular docking experiments validated the binding interaction patterns and structural behaviors of screened flavonoid compounds within the active region of the Ewing sarcoma protein (EWS). Furthermore, pharmacogenomics analysis was used to investigate potential drug interactions with Ewing sarcoma-associated genes. Finally, molecular dynamics simulations were used to investigate the stability of the best selected docked complexes. Taken together, daidzein, kaempferol, and genistein exhibited a result comparable to ifosfamide in the proposed in silico study and can be further analyzed as possible candidate compounds in biological in vitro studies against ES.

## 1. Introduction

Ewing sarcoma (ES) is the second-most common primary bone tumor that is highly malignant and has a peak incidence in the second decade of life [1]. It affects 2.6 and 2.8 children per million in the United States and Germany, respectively [2]. It is the most prevalent bone sarcoma in children and adolescents (peaking in the second decade). It originates from either neural crest cells or mesenchymal stem cells [2]. Depending upon the location of the tumor in the body, many variants of Ewing sarcoma exist, including extraosseous bone sarcomas (occurring outside of bones), skin tumors, and peripheral primitive neuroectodermal tumors (pPNET). ES typically develops in the femur, shoulder blades, ribs, and pelvic area [3]. Previous research has found that tumors most commonly arise in the tubular bones of the extremities (46%), mostly in the lower extremities, followed by the pelvis (25%), trunk comprising ribs and spinal trunk (22%), and other locations (6%) [1]. The 5-year overall survival (OS) rate for patients with localized ES has grown to 65%, due to the use of surgery, chemotherapy, and radiation [4].

EWSR1 was discovered in Ewing’s sarcoma and neuroectodermal malignancies as a translocation-generated fusion gene between EWSR1 and FLI1 (Friend leukemia integration 1 transcription factor) [5]. EWSR1 has recently been identified as a ‘hybrid’ gene implicated in several mesenchymal tumor translocations, with data indicating that it may be translocated and fused with a variety of partner genes, including EWSR1-FLI1 t(11;22) (q24;q12) and EWSR1-ERG in Ewing’s sarcoma [6], EWSR1-WT1 in desmoplastic small round cell tumors [7], EWSR1-CREB in angiomatoid fibrous histiocytoma [8], EWSR1-DDIT3 in myxoid liposarcoma [9], and EWSR1-ATF1 in clear-cell sarcoma-like tumors of the gastrointestinal tract [10]. Pathogenesis is caused by a balanced translocation of the EWS gene, which produces fusion proteins that code for chimeric transcription factors that promote cell proliferation. The most common fusion protein is EWS-FLI1 [11,12]. The EWS-FLI1 domain’s N-terminus permits EWS/FLI1 to link to RNA polymerase II and engage the barrier to the auto-integration factor complex. Moreover, the C-terminus of EWS-FLI1 maintains FLI1’s DNA-binding domain and specifically interacts with the ACCGGAAG central sequence. EWS-FLI1 primarily binds to GGAA-repetitive areas, resulting in an association between GGAA microsatellites, EWS-FLI1 binding, and target gene expression [13].

Herbs have long been utilized as traditional medicine and include a wide range of phytochemical components such as terpenoids, phenols, lignins, stilbenes, tannins, flavonoids, quinones, coumarins, alkaloids, amines, betalains, and certain other metabolites [14]. Flavonoids are low molecular weight phenolic compounds present in a broad range of plant species [15]. Flavonoids have been demonstrated to have a variety of biological features, including anti-cancer, anti-biological, anti-inflammatory, anti-mutagenic, anti-oxidant, anti-allergic, and anti-viral activity [16,17]. Flavonoids have been extensively studied for their anti-cancer properties [18]. This study aims to investigate in silico the binding of various flavonoid compounds to the EWS protein and examine their associations with the ES.

## 2. In Silico Methodology

### 2.1. Protein Structure Retrieval

The RNA recognition motif of the EWS protein, which has the PDBID: 2CPE (https://www.rcsb.org/structure/2CPE (accessed on 15 October 2022)), was assessed from the Protein Data Bank (PDB), and its energy was minimized using the UCSF Chimera 1.10.1 [19]. VADAR 1.8 (http://vadar.wishartlab.com/ (accessed on 15 October 2022)), an online server, was employed for quantitative protein structure evaluation of the Ewing sarcoma protein, composed of α-helices, β-sheets, coils, and turns. The Discovery Studio Client [20] was utilized to explore the 3D protein structure and to compute Ramachandran graphs.

### 2.2. Selection of the Binding Pocket

The position of a ligand in the protein’s holo-structure most likely determines the binding pocket of targeted protein and channels [21]. The active binding site residues were selected from previously published data [13] and identified using Discovery Studio and UCSF Chimera 1.10.1.

### 2.3. Ligand’s Preparation

Flavonoids are being used against various diseases such as cancer, viral diseases, lung cancer, anti-osteoporosis, atherosclerosis, bone cancer, and Alzheimer’s disease [16,18,22,23,24,25,26]. Recently, flavonoids have also been observed to be potent against osteosarcoma and in bone regeneration [27,28]. Flavonoids have several subgroups which include flavonols, flavones, flavanones, isoflavones, and anthocyanidins [26,29]. The 3D structures of the representative compounds from all of these subclasses were selected (on the basis of their activity against carcinogenesis) and downloaded from PubChem, and further minimized by Discovery Studio and PyMol [30]. Moreover, the 3D structure of ifosfamide as a reference compound [31] was also accessed from PubChem and minimized for comparative molecular docking studies.

### 2.4. Molecular Docking

Molecular docking is the most widely used method for evaluating the interactions and conformations of ligands with target proteins [32]. For instance, it is feasible to anticipate the association strength or binding affinity between two molecules based on preferred orientation by using scoring algorithms [33]. The CDOCKER module of Discovery Studio was employed to perform molecular docking of flavonoids to EWS. The attribute of the binding pocket sphere was modified as (X = 8.6488, Y = 3.7819 and Z = 2.6209) and the radius value was adjusted to 6.6378 [13] for a better conformational position in the active region of the target protein. The ligands (flavonoids) were docked individually to EWS with the default orientation and conformation 10/10. Consequently, the top hits were chosen as 04. The lowest binding energy values (in kcal/mol) were used to predict the docked complexes. The top four docked complexes and ifosfamide as a standard drug were represented graphically in three dimensions (3D) using UCSF Chimera 1.10.1 [19] and Discovery Studio Client.

### 2.5. Pharmacogenomics Analysis

The Drug Gene Interaction Database (DGIdb) (https://www.dgidb.org/ (accessed on 25 October 2022)) and the Disease gene network (DisGeNET) (https://www.disgenet.org/ (accessed on 25 October 2022)) were used to acquire a probable list of different disease-associated genes in order to create the pharmacogenomics network model for the top ten selected drugs. In addition, a thorough review of the literature was carried out of all anticipated genes to determine their role in ES. Furthermore, ES-associated gene clumps were sorted, and the remaining disease-associated genes were removed from the data set.

### 2.6. Molecular Dynamics Simulations

The parameters and the protocol of simulations were retrieved from already published data [34] for the 50 ns MD simulation experiment of top docked complexes. The top four complexes daidzein–EWS, genistein–EWS, kaempferol–EWS, and quercetin–EWS, which had the lowest docking energies and good correlation with ES, were subjected to molecular dynamics simulations. Furthermore, the ifosfamide drug was also subjected to MD simulation for a comparative study. The GROMACS program (version 2019.3 for Linux) was used to examine the structural behavior of protein and ligand complexes [35]. The CHARMM-GUI server’s solution builder protocol (www.charm-gui.org (accessed on 25 October 2022)) was used to generate the CHARMM36 force field, and the same interface was used to construct input files for MD simulations in GROMACS [36]. The TIP3P 3-point water model was utilized to solvate the system, a cubic box with periodic boundary conditions. Counterions were added until the system was neutralized. The Verlet algorithm, with a cut-off radius of 10 Å for electrostatic and van der Waals interactions, was employed while the LINCS algorithm was used to constrain the bond lengths during the simulations. Furthermore, the Particle Mesh Ewald (PME) method was used to calculate electrostatic interactions. The solvated systems were subjected to the steepest descent energy minimization approach. Following that, systems went through two phases of equilibration. Systems were first brought into equilibrium under the constant temperature, constant volume (NVT) condition and then under the constant temperature, constant pressure (NPT) condition. The CHARMM-GUI includes a Python script for converting GROMACS topology (top) and parameter (itp) files for MD simulations in GROMACS. To execute MD simulations in GROMACS, a 2 fs time step was used, and the coordinates were recorded every picosecond for further analysis.

## 3. Results and Discussion

**Figure 1:**
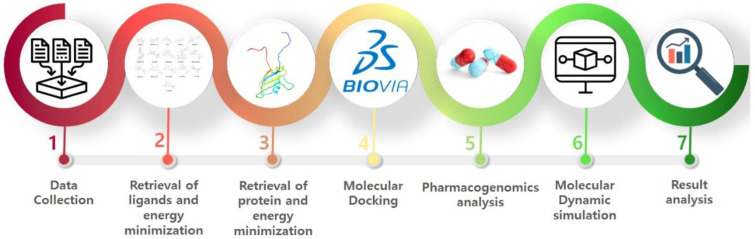
Workflow diagram of research work.

The workflow of the proposed research work is depicted in Figure 1. Seven steps were carried out to predict and observe the interactions of flavonoids with EWS protein, which included the retrieval of best flavonoids, retrieval of protein, molecular docking, pharmacogenomics analysis, and MD simulation studies.

### 3.1. Structural Analysis of the EWS Protein

**Figure 2:**
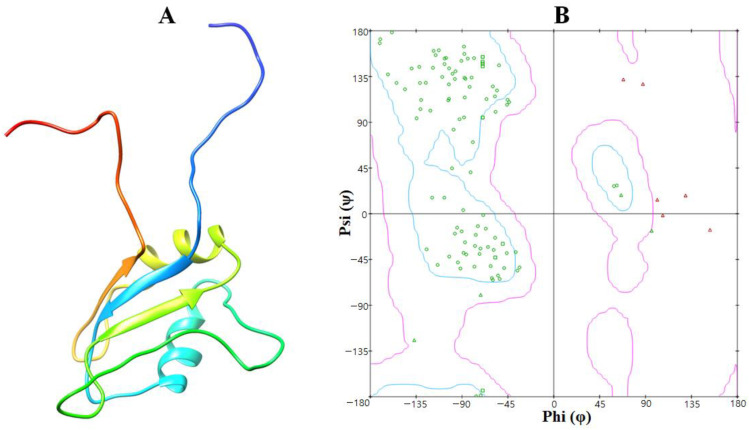
(**A**,**B**). The 3D structure of the EWS protein is on the left side mentioned as (**A**) while the computed Ramachandran plot is on the right side mentioned as (**B**). Ramachandran revealed the polypeptide backbone rotations around the bonds between N-Ca (named Phi(φ)) and Ca-C (named Psi(ψ)).

The EWS protein belongs to the class of hydrolases and is made up of 113 amino acids forming a single chain. Loops, α-helices, and β-sheets occur in the overall protein structure. Two twisted loop structures were identified at the EWS protein’s terminal regions and the core binding cavity of the helices (Figure 2). Furthermore, a VADAR 1.8 structural study revealed that EWS is made up of 25% α-helices, 30% β-sheets, 43% coils, and 20% turns. According to the Ramachandran plots, 82.0% of amino acids occur in the favored region while 98.2% of residues were in the allowed zone of dihedral angles phi (φ) and psi (ψ).

### 3.2. The Binding Pocket Analysis

**Figure 3:**
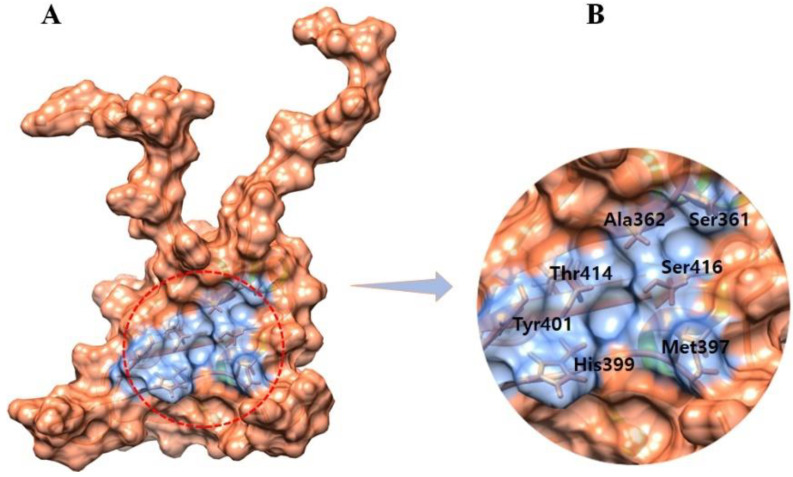
(**A**,**B**) Binding pocket surface and active binding residues are highlighted in light blue color while the whole protein surface is colored brown.

In addition to its shape and location inside a protein, a binding pocket’s function is determined by the collection of amino acid residues that surround it [21]. The binding pocket residues of EWS were retrieved from an already published research article [13] and selected as Ser361, Met397, Ala362, His399, Tyr401, Thr414, and Ser416 (Figure 3).

### 3.3. Ligand’s Preparation

**Figure 4:**
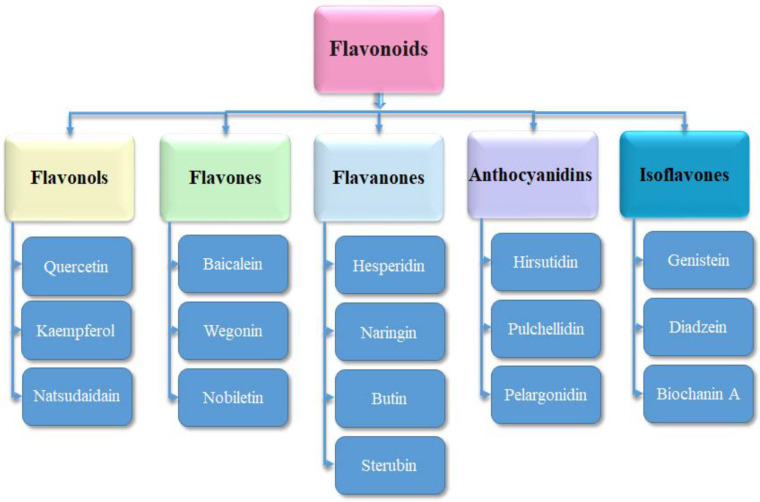
The subclasses of flavonoids and selected representative compounds from these subclasses.

**Figure 5:**
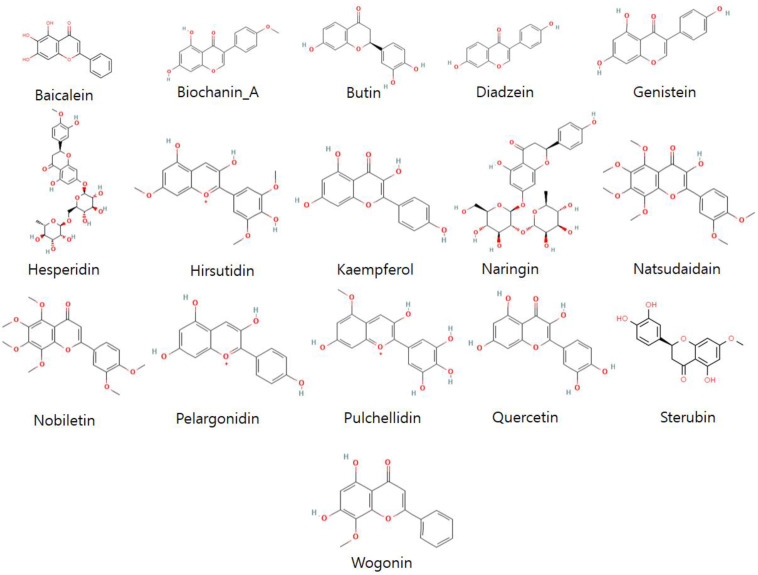
The structural assessment of 2D structures of screened flavonoids for molecular docking to EWS.

Flavonoids have several favorable biochemical, anti-cancer, anti-oxidant, anti-allergic, and anti-inflammatory characteristics against multiple diseases such as carcinogenesis, osteosarcoma, lung cancer, osteoarthritis, bone cancer, and Alzheimer’s disease (AD) [16,18,22,23,37]. The 3D structures of representative compounds (from flavonoid subclasses) (Figure 4) and their activities against biological assays were obtained through the PubChem database of chemical molecules. Further, energy minimization of these compounds was carried out by visualizing in Discovery Studio and PyMOL. The ligands were evaluated on structural (2D, 3D) analyses and prepared for future molecular docking studies (Figure 5).

### 3.4. Molecular Docking Analysis

All screened compounds (flavonoids) docked to EWS were examined independently and scored based on the minimal docking energy values and their interaction patterns (Table 1). The lowest binding energy values and ligand interaction patterns were utilized to determine the top ten flavonoid compounds. The ten corresponding ligands had good binding energy values and were bound to the target protein’s active region.

### 3.5. Pharmacogenomics Analysis

Through pharmacogenomics analysis, the respective top ten drugs with strong binding affinity and low docking energy were further examined. In order to achieve optimum efficacy with minimum side effects, pharmacogenomics strives to be a reasonable method to optimize medication therapy with regard to the genotype of the patients [38]. Therefore, a couple of pharmacogenomics databases were employed to estimate the potential relationships between the genes of the tested compounds and their associations with diseases. Based on interaction score values, the predicted genes for the compounds were sorted.

The flavonoid compounds that exhibit high interaction scores with genes involved in Ewing sarcoma are displayed in Table 2. Daidzein is associated with five genes with interaction scores ranging from 3.25 to 0.05. The IBSP gene has a higher interaction score and is involved in the malignant neoplasm of bone. Furthermore, the LIF gene, which has an interaction score of 0.59, is directly involved in ES. Kaempferol also exhibits an association with five genes, and all these five genes are involved in childhood neoplasm and carcinogenesis. Moreover, genistein is associated with six genes with interaction scores ranging from 2.47 to 0.62, and each of these genes is involved in osteosarcoma bone cancer and childhood lymphoma. Additionally, quercetin, baicalein, and wogonin are associated with three genes, two genes, and one gene, respectively, with interaction scores ranging from 0.78 to 0.13, and all these genes are involved in childhood blastoma and osteosarcoma of bones.

### 3.6. Hydrogen Bond Interaction Analysis

The top four compounds which have the lowest energies of docking to the EWS protein and best associations (computed from the pharmacogenomics analysis) with genes involved in Ewing sarcoma were further analyzed for their hydrogen bond pattern analysis.

### 3.7. Daidzein

**Figure 6:**
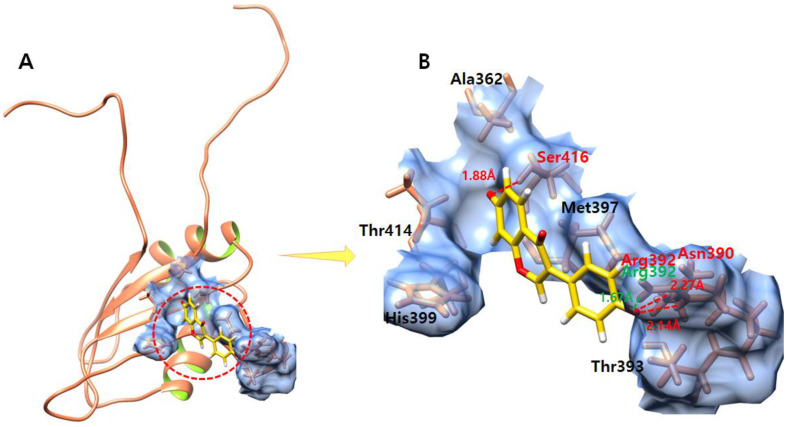
(**A**,**B**) show the daidzein–EWS complex. (**A**) illustrates the global structure of the complex, and (**B**) focuses on the binding pocket. The salt bridges and hydrogen bonds formed in the docked complex are shown in green and red color, respectively. The EWS protein is colored in coral, and helix interiors are colored in chartreuse green while the surface of the binding pocket is colored in light blue.

The daidzein compound, which exhibits the lowest interaction energy in molecular docking and high association with genes involved in ES, is confined in the active binding pocket of the EWS protein (Figure 6). The daidzein–EWS-docked complex shows that one oxygen atom of daidzein forms a hydrogen bond with Ser416 with a bond length of 1.88 Å, and the other oxygen atom of daidzein makes one hydrogen bond with Asn390 with a bond length of 2.27 Å. Furthermore, an oxygen atom forms a hydrogen bond and a salt bridge with Arg392 with bond lengths of 2.14 Å and 1.67 Å, respectively.

### 3.8. Kaempferol

**Figure 7:**
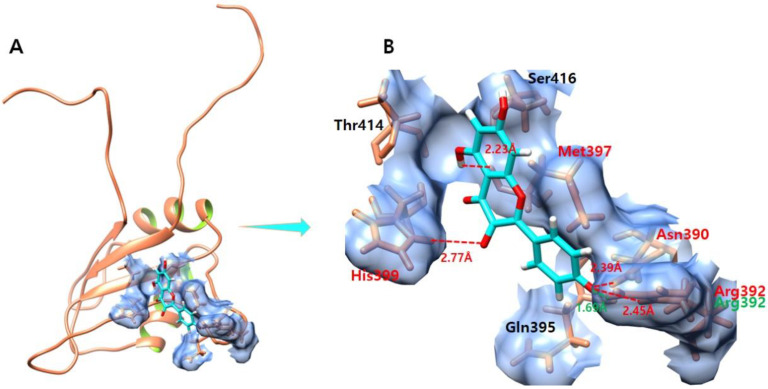
(**A**,**B**) shows the kaempferol–EWS complex. (**A**) illustrates the global structure of the complex, and (**B**) focuses on the binding pocket. The salt bridges and hydrogen bonds formed in the docked complex are shown in green and red color, respectively. The EWS protein is colored in coral, and helix interiors are colored in chartreuse green while the surface of the binding pocket is colored in light blue.

The ligand–protein docking analysis of kaempferol shows that the ligand is confined within the active region of the target protein as shown in Figure 7. The kaempferol–EWS-docked complex creates four hydrogen bonds and one salt bridge which involve Met397, His399, Asn390, and Arg392 residues. The oxygen atom of kaempferol forms a hydrogen bond with Met397 with a bond length of 2.23 Å. Another oxygen atom forms a hydrogen bond with His399 with a bond length of 2.77 Å. Furthermore, another oxygen atom of kaempferol forms a hydrogen bond and a salt bridge with Arg392 with bond lengths of 2.45 Å and 1.69 Å, respectively. Additionally, the other oxygen atom of kaempferol forms a hydrogen bond with Asn390 with a bond length of 2.39 Å.

### 3.9. Genistein

**Figure 8:**
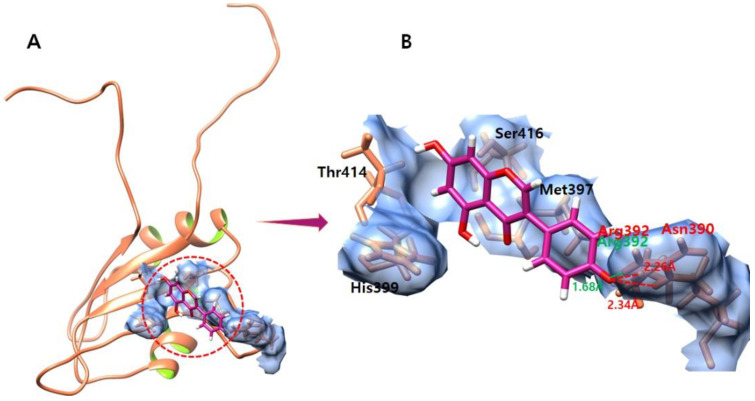
(**A**,**B**) show the genistein–EWS complex. (**A**) illustrates the global structure of the complex, and (**B**) focuses on the binding pocket. The salt bridges and hydrogen bonds formed in the docked complex are shown in green and red color, respectively. The EWS protein is colored in coral, and helix interiors are colored in chartreuse green while the surface of the binding pocket is colored in light blue.

The ligand–protein docking analysis of genistein shows that the ligand binds within the active region of the target protein as shown in Figure 8. The genistein–EWS-docked complex forms two hydrogen bonds and one salt bridge against the residues Asn390 and Arg392. The oxygen atom of genistein forms a hydrogen bond against Asn390 with a bond length of 2.26 Å. Furthermore, the other oxygen atom of genistein forms a hydrogen bond and a salt bridge against Arg392 with bond lengths of 2.34 Å and 1.68 Å, respectively.

### 3.10. Quercetin

**Figure 9:**
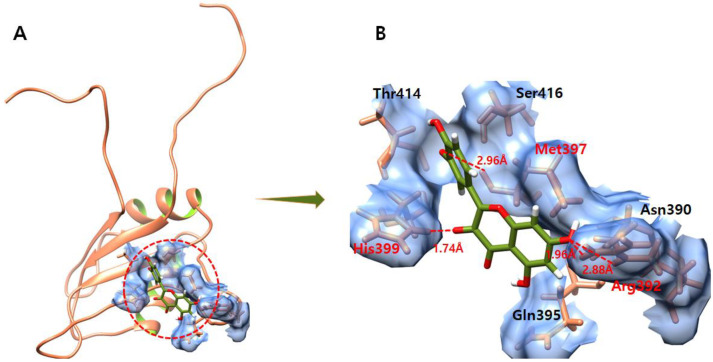
(**A**,**B**) show the quercetin–EWS complex. (**A**) illustrates the global structure of the complex, and (**B**) focuses on the binding pocket. The salt bridges and hydrogen bonds formed in the docked complex are shown in green and red color, respectively. The EWS protein is colored in coral, and helix interiors are colored in chartreuse green while the surface of the binding pocket is colored in light blue.

The ligand–protein docking analysis of quercetin shows that ligand fits well within the active region of the target protein as shown in Figure 9. The quercetin–EWS-docked complex forms four hydrogen bonds with residues Met397, His399, and Arg392. The oxygen atom of genistein forms a hydrogen bond with Met397 with a bond length of 2.96 Å. Another oxygen atom makes a hydrogen bond with His399 with a bond length of 1.74 Å. Furthermore, another oxygen atom of quercetin forms two hydrogen bonds with the same Arg392 with bond lengths of 2.88 Å and 1.96 Å.

### 3.11. Ifosfamide

**Figure 10:**
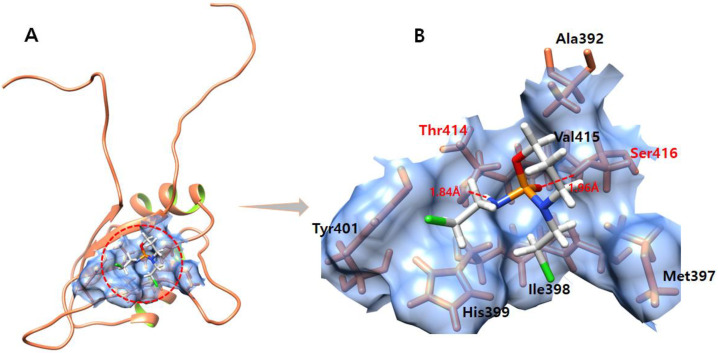
(**A**,**B**) show the ifosfamide–EWS complex. (**A**) illustrates the global structure of the complex, and (**B**) focuses on the binding pocket. The hydrogen bond formed during molecular docking is shown in red color. The EWS protein is colored in coral, and helix interiors are colored in chartreuse green while the surface of the binding pocket is colored in light blue.

The ifosfamide–EWS-docked complex shows that ligand fits well within the active region of the target EWS (Figure 10). The docked complex forms two hydrogen bonds with Ser416 and Thr414 residues. The oxygen atom of ifosfamide forms a hydrogen bonds with Ser416 with a bond length of 1.98 Å. Furthermore, another hydrogen atom produces a hydrogen bond with Thr414 with a bonding distance of 1.84 Å.

### 3.12. Molecular Dynamics Simulations

The top four complexes daidzein–EWS, genistein–EWS, kaempferol–EWS, and quercetin–EWS, which exhibited the lowest docking energies and good correlation with ES, were subjected to molecular dynamics simulations in comparison to ifosfamide.

### 3.13. Root-Mean-Square-Deviation

**Figure 11:**
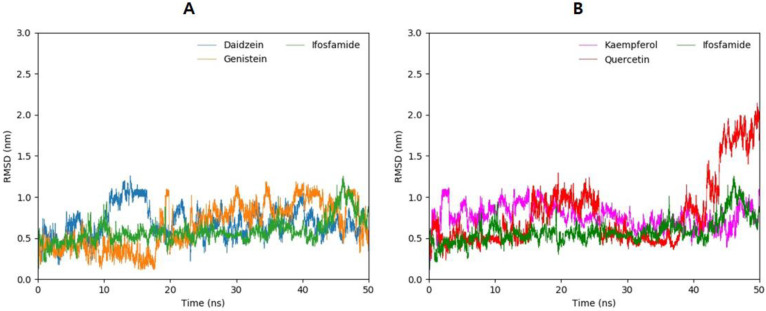
(**A**,**B**). The RMSD values for daidzein (blue) and genistein (orange) are compared with ifosfamide (green) in (**A**). Kaempferol (magenta) and quercetin (red), in comparison with ifosfamide (green), are predicted in (**B**) during the 50 ns MD simulations.

To evaluate the flexibility and overall stability of the docked complexes, 50 ns long MD simulations using GROMACS were conducted. The fluctuations of ligands inside the active site of the EWS protein were determined by the Root-Mean-Square-Deviation (RMSD) from the MD trajectories. Figure 11A,B shows the plots of the RMSD of ligands for different flavonoid–EWS protein complexes during the simulation. The daidzein molecule, which has the lowest molecular docking score and good association scores with genes that directly involved in ES, showed small fluctuations between 10 ns to 15 ns and increased its RMS deviation values to σ = 1.1, after which the conformation remains quite stable within the active site of EWS throughout the whole simulation time (Figure 11A). The stabilization of the conformation of daidzein might be due to the multiple interactions such as salt bridge formation, hydrogen bonds, and hydrophobic interactions as depicted in molecular docking. The RMSD of genistein manifests sustained confirmation at the beginning of the MD simulation with a low RMSD value but starts to unsustain after 16 ns of simulations (Figure 11B). Moreover, the RMSD values increase but the fluctuation pattern of the RMSD is maintained between σ = 0.75 and σ = 1.1 during the 50 ns time span of MD simulations. Kaempferol, which showed lower binding energy than genistein and quercetin, and multiple hydrogen bonds and salt bridge formation in molecular docking following daidzein, shows stable RMSD values between σ = 0.5 and σ = 1.1 throughout the 50 ns time span of MD simulations (Figure 11B). The quercetin molecule shows higher fluctuations than the others, and it exhibits stable conformation until 10 ns and gets higher RMSD values σ = 1.0 from 15 ns to 30 ns. The RMSD values are stabilized again for a short period of time from 25 ns to 37 ns, and then the graph fluctuates highly (Figure 11B). Additionally, MD simulation for ifosfamide, a known drug against ES, was carried out for comparative analysis. Ifosfamide exhibits lower RMSD values and fewer fluctuations in comparison with flavonoids. Only one peak at 47 ns can be seen for ifosfamide. However, flavonoids manifest comparable results to ifosfamide as shown in Figure 11A,B.

### 3.14. Hydrogen Bond Plot Analysis

**Figure 12:**
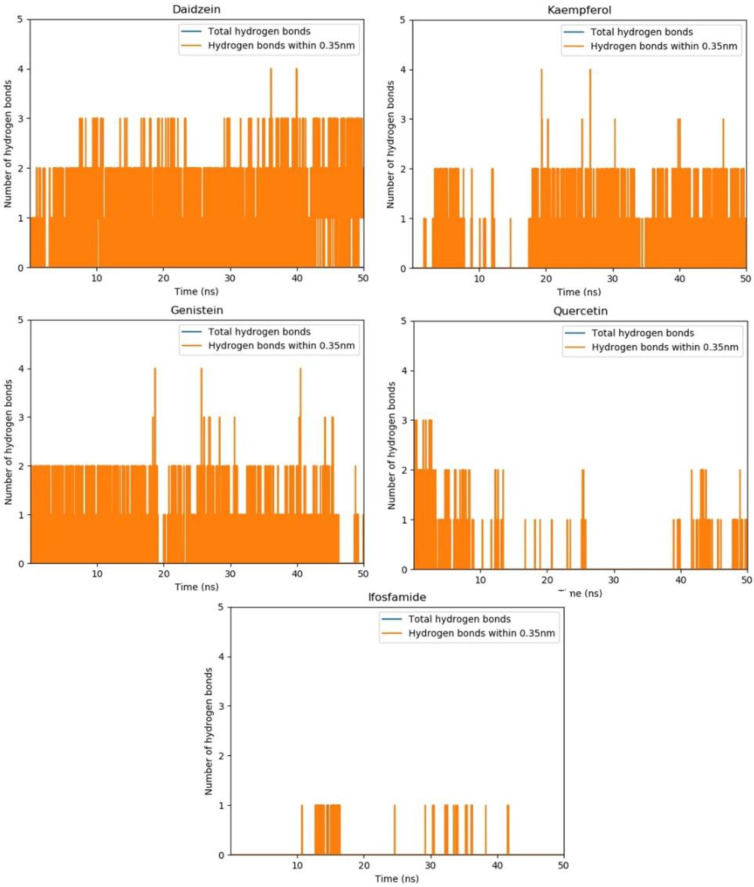
The hydrogen bond plot analysis depicts the number of hydrogen bonds shorter than 0.35 nm for daidzein, kaempferol, genistein, and quercetin in comparison with ifosfamide throughout the 50 ns MD simulations. All hydrogen bonds are tight and are shorter than 0.35, which is why no blue peak can be seen.

Hydrogen bond plot analysis distinguishes two types of hydrogen bonds based on bonding distance: hydrogen bonds with a bonding distance of less than 0.35 nm and other hydrogen bonds with lengths greater than 0.35 nm. Because of the short bonding distance, hydrogen bonds with a bond length of 0.35 nm are stronger than other hydrogen bonds. Other hydrogen bonds are assumed to be weaker since their bonding distances are greater than 0.35 nm (Figure 12).

The hydrogen bond plot analysis of daidzein depicts a high ratio of hydrogen bond formation. It shows three stronger hydrogen bonds under 0.35 nm. Additionally, the peaks corresponding to the fourth hydrogen bond can also be seen during the 50 ns time span of MD simulations. Furthermore, kaempferol and genistein also exhibit a good tendency for hydrogen bond formation during the time period of MD simulations. Both genistein and kaempferol form mostly two hydrogen bonds within the active site of the EWS protein while the peaks corresponding to the third and the fourth hydrogen bond can also be seen. All the hydrogen bonds were shorter than 0.35 nm, which indicates strong hydrogen bonding. On the other hand, quercetin and ifosfamide manifest a low tendency for hydrogen bond formation compared with daidzein, kaempferol, and genistein. Quercetin shows two and three hydrogen bonds at the beginning of simulations. After 15 ns, it starts fluctuating, and the pattern of hydrogen bonds disappears. Then, after 40 ns, again one or two hydrogen bonds can be observed. The ifosfamide drug, which shows the most stable RMSD values, has the lowest tendency for hydrogen bond formation. This could be due the fact that the flavonoids have more hydrogen bond donor and acceptor capabilities compared with ifosfamide.

### 3.15. Interaction Energy Analysis

**Figure 13:**
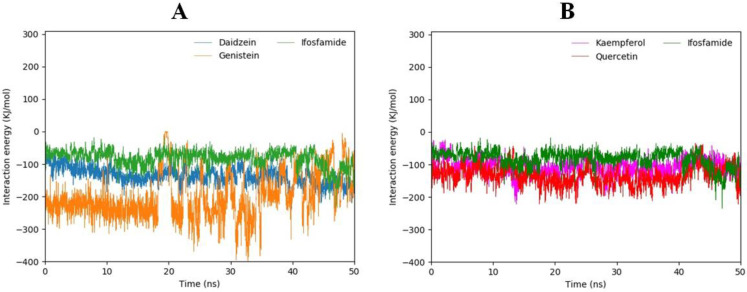
(**A**,**B**). The computed total interaction energies of daidzein (blue) and genistein (orange) in comparison with ifosfamide (green) during MD simulations are shown in graph (**A**) while graph (**B**) shows the computed total interaction energies of kaempferol (pink) and quercetin (red) compared with ifosfamide (green).

Along with the hydrogen bond visualization and RMSD analysis, the interaction energies of all four compounds docked to the EWS protein were calculated in comparison with ifosfamide during a 50 ns MD simulation to assess the interaction energy score values of the docked complexes. The interaction energy is composed of two terms: electrostatic (Coulombic) interaction energy and Lennard–Jones interaction energy, with their sum representing the total interaction energy. According to the interaction energy analysis, genistein showed the lowest total interaction energy followed by daidzein, quercetin, and kaempferol (see Table 3), but ifosfamide manifested the highest interaction energy, as predicted in molecular docking studies. Furthermore, the total interaction energy had also been plotted for all four compounds against ifosfamide (Figure 13A,B). Daidzein and genistein compared with ifosfamide are shown in graph A while kaempferol and quercetin, in comparison with ifosfamide, are predicted in graph B. Graph A shows that the interaction energy of genistein is highly fluctuating towards the lowest interaction energy while daidzein depicts a more stable graph compared with ifosfamide. Graph B shows stable plots for kaempferol, quercetin, and ifosfamide. Therefore, the genistein compound exhibits the lowest total interaction energy while daidzein, kaempferol, and quercetin have stable and lower total interaction energies in comparison with ifosfamide.

### 3.16. Binding Mode Analysis

**Figure 14:**
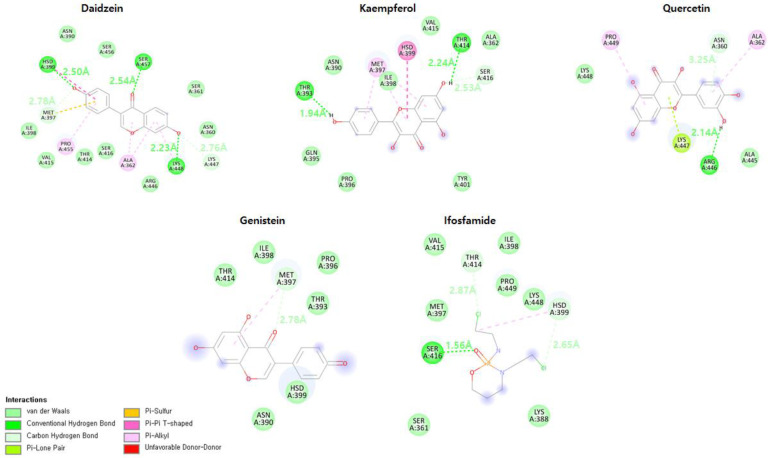
The binding patterns of daidzein, kaempferol, genistein, and quercetin compounds to the EWS protein after 50 ns MD simulations are shown and compared with the ifosfamide–EWS complex.

The mechanism of binding of flavonoid compounds to the EWS protein has been further investigated, and the stability of the docked complexes have been examined during MD simulations over a time period of 50 ns. After 50 ns of MD simulations, snapshots of all five complexes were acquired, and the binding interaction patterns were visualized using Discovery Studio and UCSF Chimera tools [19,20]. The daidzein molecule, which has the lowest docking energy, maintained three conventional hydrogen–hydrogen bonds with His399, Ser457, and Lys448 and two carbon–hydrogen bonds with Lys447 and Met397 (Figure 14). Kaempferol formed two conventional hydrogen–hydrogen bonds with Thr414 and Thr393 and one carbon–hydrogen bond with Ser416while genistein has only one carbon–hydrogen bond with a bond length of 2.78 Å. Genistein manifests mostly two hydrogen bonds until 47 ns; however, at 50 ns, it predicts only one hydrogen bond in a hydrogen bond plot analysis. Quercetin maintained one conventional hydrogen–hydrogen bond with Arg446, one carbon–hydrogen bond with Asn360, and a lone-pair–π bonding with Lys447. In comparison, the ifosfamide drug formed one conventional hydrogen bond with Ser416 and two carbon–hydrogen bonds with His399 and Thr414. These findings clearly indicate that hydrogen bond formation in the active site leads to the stability of ligand–EWS protein interactions.

## 4. Conclusions

The current study evaluates the therapeutic properties of known flavonoids against ES using flavonoid compound screening, molecular docking, pharmacogenomics, and MD simulations. The docking studies and pharmacogenomics assessments have shown that, from the group of 16 flavonoids, four compounds were most active and showed good results compared with the rest. The detailed pharmacogenomics and extensive data mining showed that these four compounds are associated with various genes linked to ES, bone cancer, and childhood carcinoma. Additionally, MD simulation results have shown that three of these four compounds presented better profiles with respect to their RMSD, hydrogen bond plot, interaction energy, and the binding mode analysis; moreover, mostly stable behavior was observed for these three docking complexes. Overall, it has been concluded that three flavonoids—daidzein, kaempferol, and genistein—exhibited a better therapeutic profile against ES in comparison with other flavonoids. All these flavonoids have low toxicity and more capabilities of forming hydrogen bonds as donors and acceptors in comparison with other compounds. Therefore, daidzein, kaempferol, and genistein have the potential for being used in the treatment of ES after detailed in vitro and clinical assessments in the future.

## Figures and Tables

**Table 1:** The docking energy values (in kcal/mol) of flavonoids docked to Ewing sarcoma protein.

No	Flavonoids	CDOCKER Energy (kcal/mol)	CDOCKER Interaction Energy (kcal/mol)
1	Daidzein	−48.34	−52.15
2	Kaempferol	−44.60	−56.40
3	Genistein	−39.86	−45.43
4	Quercetin	−30.14	−39.42
5	Pelargonidin	−29.67	−47.85
6	Pulchellidin	−28.66	−44.59
7	Baicalein	−23.63	−24.15
8	Butin	−22.99	−28.45
9	Sterubin	−19.87	−25.75
10	Wogonin	−16.57	−24.84
11	Biochanin_A	−16.55	−28.38
12	Ifosfamide	−16.23	−23.50
13	Hirsutidin	−8.08	−42.58
14	Natsudaidain	−4.84	−49.36
15	Nobiletin	−0.23	−35.30
16	Naringin	0.92	−46.53
17	Hesperidin	5.97	−42.60

**Table 2:** Pharmacogenomics analysis table showing associations of flavonoids with specific genes involved in Ewing sarcoma.

Drug	Gene	Interaction Score	Disease	Reference
Daidzein	IBSP	3.25	Malignant Neoplasm of bone	[39]
	FOS	0.65	Osteosarcoma of bone	[40]
	LIF	0.59	Ewing Sarcoma	[41]
	PIK3CG	0.07	Osteosarcoma of bone	[42]
	RACGPA1	0.05	Carcinoma of lung	[43]
Kaempferol	CTDSP1	0.19	Neoplasm	[44]
	NFKB2	0.18	Carcinogenesis	[45]
	RELA	0.14	Childhood Ependymoma	[46]
	NFKB1	0.08	Childhood Lymphoma	[47]
	RACGAP1	0.05	Childhood Grade III Meningioma	[48]
Genistein	PAEP	2.47	Osteosarcoma of bone	[49]
	EPHA8	1.24	Adenocarcinoma of lung	[50]
	PTGES3	0.82	Osteosarcoma of bone	[24]
	CCNA2	0.62	Osteosarcoma of bone	[51]
	TJP1	0.62	Childhood Lymphoma	[52]
	CEL	0.62	Childhood Acute Lymphoblastic Leukemia	[53]
Quercetin	PKN1	0.78	Childhood Rhabdomyosarcoma	[54]
	GABPA	0.52	Childhood Neuroblastoma	[55]
	HSF1	0.52	Osteosarcoma of bone	[56]
Baicalein	HIF1AN	1.65	Childhood Glioblastoma	[57]
	EGLN1	0.62	Childhood Glioblastoma	[58]
Wogonin	GMNN	0.13	Childhood Burkitt Lymphoma	[59]

**Table 3:** The averaged computed interaction energies of daidzein, genistein, kaempferol, quercetin, and ifosfamide with the EWS protein during MD simulations. Electrostatic and Lennard–Jones contributions and the total energies are predicted.

Sr No	Compound	Interaction energy		Total Energy
		Coul-SR	LJ-SR	
**1**	Daidzein	−36.6801	−99.5852	−136.2653
**2**	Kaempferol	−26.2828	−79.1068	−105.3896
**3**	Genistein	−141.839	−57.418	−199.257
**4**	Pazopanib	−47.6918	−139.997	−187.6888
**5**	Quercetin	−47.2754	−87.048	−134.3234

## Data Availability

Data are contained within the article.

## Sample Availability

Data are contained within the article.
